# Out of Asia: the independent rise and global spread of fluoroquinolone-resistant *Shigella*

**DOI:** 10.1099/mgen.0.000171

**Published:** 2018-03-29

**Authors:** Hao Chung The, Stephen Baker

**Affiliations:** Enteric Infections, Oxford University Clinical Research Unit, Ho Chi Minh City, Vietnam

**Keywords:** *Shigella*, fluoroquinolone resistance, Asia, quinolone resistance determining region (QRDR), epidemiology, genomic

## Abstract

*Shigella* are ranked among the most prevalent aetiologies of diarrhoeal disease worldwide, disproportionately affecting young children in developing countries and high-risk communities in developed settings. Antimicrobial treatment, most commonly with fluoroquinolones, is currently recommended for *Shigella* infections to alleviate symptoms and control disease transmission. Resistance to fluoroquinolones has emerged in differing *Shigella* species (*S. dysenteriae*, *flexneri* and *sonnei*) since the turn of the 21st century, originating in endemic areas, and latterly spreading into non-endemic regions. Despite occurring independently, the emergence of fluoroquinolone resistance in these different *Shigella* species shares striking similarities regarding their epidemiology and resistance mechanisms. Here, we review and discuss the current epidemiology of fluoroquinolone-resistant *Shigella* species, particularly in the light of recent genomic insights.

**Impact Statement**

*Shigella* i*s* a genus of human-adapted bacterial pathogens that cause dysenteric diarrhoea (shigellosis) in developing and developed countries. A specific class of antimicrobials, known as the fluoroquinolones, is recommended for the treatment of shigellosis, but resistance to this group of antimicrobials is rising rapidly within this genus. Here, we have combined available epidemiological and high-resolution genomic data to outline common themes that define the emergence and circulation of fluoroquinolone-resistant *Shigella* species. The information gathered in this review will be useful for determining optimal shigellosis treatment regimens and to tailor public health measures for alerting, containing and preventing the future spread of these antimicrobial-resistant enteric pathogens.

## Introduction

*Shigella*, a pathogenic genus within the extensive Gram-negative family *Enterobacteriaceae*, is a major cause of diarrhoeal disease worldwide [1, 2]. The global burden of shigellosis is estimated to be 125 million cases per year, of which 160 000 lead to death [2, 3]. The disease disproportionately affects young children in low-income tropical settings, where malnutrition, inadequate sanitation and limited access to clean water appear to facilitate the transmission of the infecting organisms. The genus *Shigella* does not comprise a monophyletic group of organisms but is formed of multiple discrete *Escherichia coli* lineages, all of which harbour a signature virulence plasmid responsible for the distinctive invasive pathogenesis [4, 5]. Current serology classifies the genus into four species or serogroups (*S. dysenteriae*, *boydii*, *flexneri* and *sonnei*), which differ significantly in their epidemiology. Toxigenic *S. dysenteriae* serotype 1 (Sd1) is the causative agent of the now rare, often fatal, epidemic bacillary dysentery. *S. boydii* is only sporadically isolated from diarrhoeal cases in the Indian subcontinent [6–8]. The overwhelming majority of shigellosis cases are presently attributed to *S. flexneri* and *S. sonnei*, which predominantly circulate in developing and developed regions, respectively [9].

**Fig. 1.**
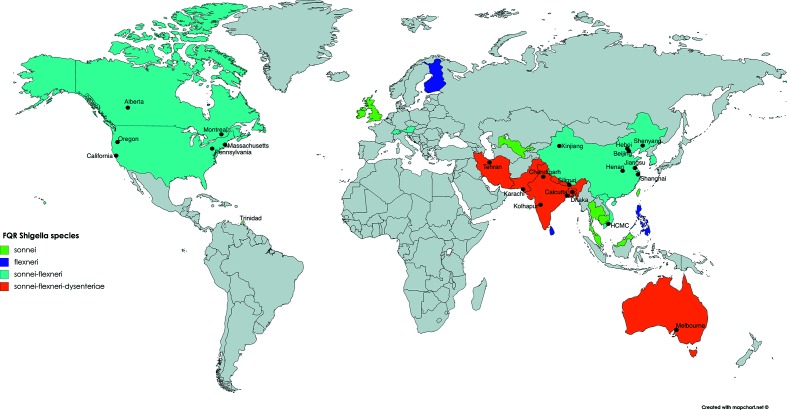
Worldwide distribution of FQR *Shigella*. Countries are coloured where different FQR *Shigella* species have been reported in the literature (see key). Black filled circles indicate specific regions where FQR *Shigella* have been isolated. Countries with no information or have not reported isolation of FQR *Shigella* are coloured grey. HCMC, Ho Chi Minh city.

Shigellosis usually results in profuse diarrhoea, often accompanied by mucous or bloody discharge. This clinical presentation is associated with disruption of the intestinal epithelium, which is mediated by intracellular proliferation of the infecting *Shigella*. Although the disease is self-limiting, antimicrobial treatment is recommended to prevent further complications, assist recovery and restrict faecal shedding [10, 11]. One of the most commonly prescribed groups of antimicrobials for shigellosis is the fluoroquinolones, which directly interact with the bacterial DNA gyrase (encoded by *gyrA* and *gyrB*) and topoisomerase IV (encoded by *parC* and *parE*) to inhibit functional replication and induce bacterial cell death [12]. Routine surveillance has documented dramatic increases in the frequency of fluoroquinolone-resistant (FQR) *Shigella*, estimating that resistance increased from 0.6 % in 1998–2000 to 29 % in 2007–2009 of the endemic shigellosis in Asia and Africa [9, 13] (Fig. 1). The genetic mechanism(s) underlying resistance is commonly attributed to mutations in the quinolone resistance determining region (QRDR), ultimately diminishing the interaction between the antimicrobial and its target proteins [14]. Resistance to fluoroquinolones narrows ever-dwindling treatment options, placing those who are vulnerable at the increased risk of complications and hampering the efficient management of outbreaks. These factors have placed FQR *Shigella* on the list of global priority pathogens that urgently need focused development of novel antimicrobials [15]. This review aims to summarize the epidemiology of various FQR *Shigella* species, highlighting insights provided through genome sequencing and phylogenetic reconstruction. Due to its low prevalence and research focus, *S. boydii* will be excluded from this discussion.

### *Shigella dysenteriae* 

**Fig. 2.**
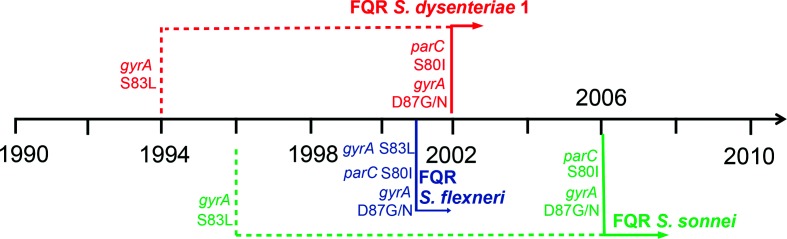
Timeline detailing the emergences of FQR *Shigella* species. The dashed lines represent the first occurrences of the initial QRDR mutation in the FQR clone if known, as described by epidemiological or genomic data. The solid lines indicate the first reports of FQR *Shigella* species as well as the QRDR mutations that became incorporated into these clones by this designated time. The presumed order of occurrence for these mutations is from top to bottom.

Resistance against fluoroquinolones had not been previously observed in Sd1 until FQR organisms were isolated during a dysentery outbreak arising in India and Bangladesh in 2002–2003 [16, 17] (Fig. 2). Molecular characterization by pulse field gel electrophoresis (PFGE) revealed that all contemporaneous FQR Sd1 isolates, causing either the outbreak or sporadic episodes across South Asia, belonged to a single clone [18–20]. However, fluoroquinolone resistance was attributed to two different QRDR mutation profiles: *gyrA*-S83L/D87G and S83L/D87N, which were associated with different geographical distributions [19]. These data suggested that the clone may have first acquired a *gyrA*-S83L mutation as early as 1994, later diverging into two FQR sub-populations, which were then characterized by differing secondary mutations. Indeed, a genomic investigation of the global phylogeny of Sd1 concluded that resistance to fluoroquinolones was acquired only once during the species’ evolutionary history, conferred by the co-occurrence of *parC*-S80I, *gyrA*-S83L and a secondary *gyrA*-D87 mutation between 1995 and 2002 [21]. This FQR clone belonged to the internationally successful lineage IV, which has witnessed at least nine independent single QRDR mutational events since the 1970s. The emergence of FQR Sd1 was followed by an abrupt decline after the outbreak, hampering routine monitoring and making the prospect of future FQR Sd1 outbreaks unpredictable [20, 22]. Information regarding resistance in alternative *S. dysenteriae* serotypes is limited, probably due to their low prevalence, even in regions where the disease was once highly endemic.

### *Shigella flexneri* 

The majority of epidemiological research on *S. flexneri* has been conducted in South Asia and China, where the pathogen’s burden remains significant. The first incidences of FQR in *S. flexneri* were documented in eastern and northern China in 2001–2002 [23, 24], and a detailed genetic screen revealed that the majority of these organisms possessed *gyrA*-S83L, *gyrA*-D87G and *parC*-S80I QRDR mutations [22]. Subsequently, a thorough examination of >2000 Bangladeshi *S. flexneri* underlined a worrying rising trend of fluoroquinolone resistance, which was almost exclusively found in serotype 2a [22]. Although it initially appeared in 2005, fluoroquinolone resistance escalated rapidly and its prevalence was >40 % of all native *S. flexneri* by 2010. These Bangladeshi isolates differed from their Chinese counterparts by a secondary QRDR mutation, harbouring *gyrA*-D87N instead of G. Furthermore, FQR *S. flexneri* with identical mutation profiles were recovered during a decade-long surveillance in Switzerland, highlighting the occurrence of fluoroquinolone resistance in non-endemic regions [25].

Routine dysentery surveillance in China has reported a steady increase of FQR *S. flexneri* of various serotypes, including 1a, 1c, 2a, 2b, 2av, 4a, 4c and X [26–31]. This observation suggests that the FQR phenotype has either emerged on numerous independent occasions across several serotypes or was acquired once, prior to subsequent intensive serotype switching events. Previous genomic studies reported that serotype conversion within a lineage is a commonly observed phenomenon for *S. flexneri* [32, 33]. Available literature provides greater support for the role of serotype switching on creating multiple FQR *S. flexneri* serotypes. Despite being present in a wide range of serotypes and locations, identical QRDR mutations have been frequently encountered in *S. flexneri* in China, encompassing *gyrA*-S83L, *gyrA*-D87G/N and *parC*-S80I. Furthermore, these mutations are commonly accompanied by an unusual mutation (*gyrA*-H211Y), which was also present in the aforementioned Bangladeshi FQR isolates [22, 28, 31, 34]. FQR *S. flexneri* from these two countries were also found to exhibit a close genetic relationship via PFGE [22]. This combined evidence indicates that spatially dispersed FQR *S. flexneri* probably belong to one dominant widespread clone, where the *gyrA*-H211Y, *gyrA*-S83L and *parC*-S80I mutations arose prior to geographical divergence. Later, a secondary mutation in *gyrA* delineated the Bangladeshi (*gyrA*-D87N) and the Chinese (*gyrA*-D87G) FQR isolates. However, the increasing isolation frequency of the *gyrA*-D87N variant in parts of China may be the result of a higher degree of trans-border dissemination from South Asia and/or the separate, indigenous emergence of a competent FQR subclone [31]. Due to the degree of genetic diversity and the complex population structures within *S. flexneri*, the true nature of such events cannot be easily measured using low-resolution molecular typing methods.

### *Shigella sonnei* 

A shift in species dominance (from *S. flexneri* to *S. sonnei*) has been observed concurrently in multiple Asian countries as they undergo rapid economic transition; this has been recorded in Bangladesh, China, Thailand and Vietnam [35–39]. This intriguing trend greatly increases the burden of *S. sonnei* worldwide, making antimicrobial resistance in this species a focal target for monitoring. Surveillance studies in developed countries have identified strong epidemiological links between FQR *S. sonnei* and a travel history to India [40, 41]. Moreover, despite disparate spatial distributions, these isolates share the same pulsotype (via PFGE) with FQR *S. sonnei* recovered in South Asia [36, 42–44]. These results suggest that contemporaneous FQR *S. sonnei* are clonal and have evolved and spread in the region before disseminating intercontinentally. Indeed, phylogenetic analysis on representative extant FQR *S. sonnei* confirmed this hypothesis, concluding that South Asia was the most likely origin of these organisms [45]. Furthermore, this study identified two distinct regional diversifications of the FQR clone out of South Asia, with one circulating in Southeast Asia and another appearing to instigate sustained transmission within Europe and America. These observations concur with frequent reports of native FQR *S. sonnei* circulating in Cambodia, Vietnam and California [46–48]. Fluoroquinolone resistance in *S. sonnei* is generally determined by the sequential accumulation of three mutations: *gyrA*-S83L, *parC*-S80I and *gyrA*-D87G [45, 49]. However, other resistance mechanisms, including differing mutations in QRDR (*gyrA*-D87N instead of D87G) and the synergy between the plasmid-mediated *qnrB* gene and *gyrA* mutations, have also been identified [50, 51]. It is of particular concern that the transmission of the FQR clone is intensified in high-risk contact networks, such as those reported among MSM (men who have sex with men) communities in non-endemic Canada and Taiwan [52, 53]. Therefore, the propagation of FQR *Shigella* should be closely monitored in MSM networks, especially in the wake of increased shigellosis incidence, HIV infection and resistance to other antimicrobials, such as azithromycin, within this high-risk group [54].

## Outlook

The presented evidence reveals striking similarities between the emergence of fluoroquinolone resistance among the discrete *Shigella* species. (1) FQR is almost exclusively determined by sequential QRDR mutations in the following order: *gyrA*-S83L, *parC*-S80I and *gyrA*-D87G/N. (2) To date, the majority of FQR isolates identified within an individual species are clonal despite their wide geographical distribution. (3) South Asia, and potentially China, serve as likely reservoirs for the rise and spread of resistant clones. These interpretations are currently deduced from genomic insights into Sd1 and *S. sonnei*, and are subjected to various confounders, including geographical bias in sample collection. However, the extensive genetic diversity within *S. flexneri* may present an alternative scenario, which will benefit from large-scale molecular epidemiology data generated through whole-genome sequencing.

The first widely used fluoroquinolone, ciprofloxacin, was introduced to clinical practice in 1987. However, resistance in *Shigella* only began to emerge in the early 2000s. The intervening period witnessed the emergence of *Shigella* exhibiting resistance to multiple antimicrobials including co-trimoxazole, ampicillin and nalidixic acid [55]. Therefore, fluoroquinolones, such as ciprofloxacin, began to be deployed more commonly to manage drug-resistant shigellosis, and its use became routine, as recommended by the World Health Organization in 2005 [11, 56]. Recent experimental and modelling work into the evolution of fluoroquinolone resistance could offer explanations for the observed pattern between the various *Shigella* species. The ordered QRDR mutations are selected in favour of those in efflux regulatory machinery due to their co-optimization for non-susceptibility (MIC levels) and fitness cost [57, 58]. For both *in vitro* generated and clinical isolates, resistance to fluoroquinolones almost exclusively commences with an initial mutation, *gyrA*-S83L. This mutation has arisen independently on multiple occasions for different *Shigella* lineages, possibly as an adaptive strategy for resistance against the first-generation quinolone, nalidixic acid [21, 32, 59]. However, the key determining factor in this stepwise evolution is the subsequent mutation, *parC*-S80I, which occurs much less frequently during evolution but gives the bacterium a significant increase in fluoroquinolone MIC and potentially a non-inferior fitness. Such a mutation is suggested to be favourably selected in abundance of mutation supply, fulfilled either by a large population size or a high mutation rate, when antimicrobial pressure is high [58]. Given that the mutation rate of the bacterium *Shigella* is relatively stable, the first scenario appears to be more plausible [21, 32, 49, 59]. High population densities in South Asia could promote extensive and sustained *Shigella* transmission, resulting in a large bacterial population. Suboptimal public health measures in the region, exemplified by the fact that only 40 % of the Indian population has access to improved sanitation [60], further amplify the transmission cycle of *Shigella*. This expansion has arisen on a backdrop of rapidly increasing fluoroquinolone use for treatment of multiple enteric and febrile diseases since the turn of this century [56, 61]. Indeed, India, with 12.9 billion units, was ranked as the world’s largest antimicrobial consumer in 2010 [62]. These contributing factors might render South Asia a unique focal point for the emergence of human-restricted FQR enteric bacteria, including *Shigella* and *Salmonella* Typhi [63].

## Conclusion

The emergence of FQR *Shigella* has been quickly followed by the expansion and, for *S. sonnei*, rapid international spread. Furthermore, co-resistance to other first-line antimicrobials, such as the macrolides and third-generation cephalosporins, is frequently identified among these bacteria [47, 50]. These antimicrobial resistance combinations present a serious public health threat for the effective treatment and management of shigellosis. It has been experimentally demonstrated that the described QRDR mutations may carry no detrimental or limited fitness cost to the resistant *Enterobacteriaceae*, even in the absence of fluoroquinolone pressure [57, 64]. Alternatively, fluoroquinolone resistance has been coupled with the successful clonal propagation of several multi-drug-resistant pathogens, including *Staphylococcus aureus*, *Klebsiella pneumoniae*, *E. coli*, *Clostridium difficile* and *Neisseria gonorrhoeae* [65, 66]. All these major FQR clones were found to harbour specific QRDR mutation combinations, indicating that these resistance genotypes induce a minimal fitness disadvantage. Although little is known about the impact of alleviating fluoroquinolone pressure on the clonal dominance of FQR bacteria in nature, we speculate that withdrawal of such pressure in clinical settings is unlikely to discontinue the dominance of FQR *Shigella* in the transmission chain. However, future research is warranted to challenge this hypothesis, as well as to develop the best practices for controlling and treating new emerging antimicrobial-resistant clones.
